# Detrimental Effects of Restricted Cage Size on Reproductive Performance, Exploration Ability, and Anxiety but Not Working Memory of Kunming Mice

**DOI:** 10.3389/fnbeh.2021.651782

**Published:** 2021-05-26

**Authors:** Wenzhen An, Ying Zhang, Aibao Zhou, Yuzheng Hu

**Affiliations:** ^1^School of Psychology, Northwest Normal University, Lanzhou, China; ^2^Center Lab, First Hospital of Lanzhou University, Lanzhou, China; ^3^Department of Psychology and Behavioral Sciences, Zhejiang University, Hangzhou, China

**Keywords:** cage size, Kunming mice, reproductive ability, anxiety, working memory

## Abstract

A suboptimal housing environment such as small cage size can adversely influence many aspects of the biology of laboratory animals including their response in behavioral tests. However, the effect of cage size on the mental and physical conditions of Kunming mice, which have been widely used to develop models of depression, anxiety, and many other diseases in China, are still far from clear. The purpose of this study was to investigate the effects of cage size on reproductive ability, exploratory behavior, anxiety, and working memory of Kunming mice. Two cage sizes were used, including a standard cage (20 × 30 × 25 cm^3^) and a restricted cage (10 × 20 × 25 cm^3^). The results revealed that compared with mice in the standard cages, mice in the restricted cages showed: (I) a decreased delivery rate of dams (*P* < 0.05) and a lower survival rate of offspring (*P* < 0.05), specifically in females (*P* < 0.05); (II) a decreased exploratory behavior (*P* < 0.01) and an increased anxiety level (*P* < 0.01); and (III) higher working memory in the *T*-maze test (*P* < 0.05). These results indicated that a restricted cage size has detrimental effects on the reproductive ability and anxiety level, but its effect on cognitive ability is complex and warrants further study. In short, these results provide empirical evidence for better practices in caring for Kunming mice, with some cautions about the effects of cage size on behavioral tests.

## Introduction

Nowadays, much attention is being paid to the welfare of laboratory animals (Reeb-Whitaker et al., 2001; Gonder and Laber, 2007), and the conditions of keeping laboratory animals have been the focus of several studies, as environmental factors such as cage size may interfere with the results of behavioral tests (Loo et al., 2001; Mcglone et al., 2001; Wolfer et al., 2004; Julia et al., 2008). For example, previous studies have shown significant effects of cage size on reproductive performance (Julia et al., 2008; Whitaker et al., 2009), aggressiveness level (Loo et al., 2001), anxiety level (Bellei et al., 2011), and exploration and cognitive abilities (Forsyth and Young, 2007; Julia et al., 2008; Line et al., 2010). Therefore, animal welfare, including cage size, is critical to the reliability of research using laboratory animals.

In previous studies, four indicators, which include litter size, number of pups that survive to weaning age, average pup weight at 21 days after birth, and number of days between litter births, have been used to characterize the reproductive performance of mice (Whitaker et al., 2009). Studies have shown that cage size is important to animal reproduction (Aro and Adejumo, 2010; Koketsu and Iida, 2017), but larger cages are not necessarily more conducive to an animal’s reproductive performance (Whitaker et al., 2009). Most studies agree that cage size affects the spontaneous activity, aggression, anxiety, and cognition (Loo et al., 2001; Steyermark and Mueller, 2002). In fact, mice in larger or smaller cages are all becoming more aggressive (Loo et al., 2001; Gupta et al., 2007; Buijs et al., 2011), but few studies mention the effect of cage size on cognition, especially working memory. Further, it has been shown that the effects of cage size upon animal reproductive performance and behaviors vary across species (Nicol et al., 2006; Buijs et al., 2009; Moreira et al., 2010). Therefore, the optimal cage size should be determined specifically for a given species as well as different strains of a species.

Similar to C57BL mice, Kunming (KM) mice are laboratory animals that have been widely used in scientific research, partially because they are disease resistant and environment-adaptable with relatively high reproductive performance and survival rate (Haitao et al., 2009). Especially in China, most biomedical studies employing mice have been carried out using KM mice (Pang et al., 2015; An W. et al., 2017; Hou et al., 2017; An et al., 2018). As different strains of mice exhibit different physiological characteristics, they may respond differently to environmental factors such as the cage size (Wang et al., 2012). Moreover, increasing numbers of studies use KM mice for developing models of depression, anxiety, and other diseases (An D. et al., 2017; Gao et al., 2017; Xianwen et al., 2018). However, there are no clear reports relating to the effects of cage size on the behavior of KM mice. The aim of the present study was to evaluate the effects of cage size on the reproductive ability, exploratory behavior, anxiety, and cognition of KM mice. Delivery rates, litter size, survival rates, and ratio of males to females were used to evaluate the reproductive ability of mice. The open-field test (OFT) was used to evaluate exploratory behavior and anxiety levels, whereas the *T*-maze test was used to characterize working memory, a critical capability that contributes to cognition.

## Materials and Methods

### Animals

Ninety mice (60 females of 28 ± 2 g and 30 males of 30 ± 2 g) were purchased from the Laboratory Animal Center of Lanzhou University (Lanzhou, China) and used in the reproductive performance test, and their offspring were used in the behavioral tests described below.

All mice were kept in a temperature- and humidity-controlled environment with a 12-h light/12-h dark cycle (lights on at 7:00 a.m.) and enough food and water. All animal studies and experimental procedures were approved by the Animal Care and Use Committee of the animal facility at Northwest Normal University.

### Cages

Cages were available in two sizes—standard (20 × 30 × 25 cm^3^) and restricted (10 × 20 × 25 cm^3^; Suzhou Fengqiao Purification Equipment Company Limited China). All cages met the animals’ basic needs, containing enough corn cob bedding and tissue papers for nesting (Jiangsu Xietong Biological Engineering Company Limited, Nanjing, China).

### Methods

Reproductive performance: one male mouse was paired with two females in a standard cage. Twenty-four hours later, pregnant mice were randomly and equally assigned to standard cages or restricted cages, with one pregnant mouse per cage. Among 60 females used to raise offspring, eight were not pregnant. The remaining 52 mice were randomly assigned to either the standard cage group or restricted cage group, with 26 in each group. The gestation and lactation period lasted about 50 days. Once every 2 days, feed and water were replaced and the cages were cleaned, except that the cages were not cleaned within 10 days after delivery, and stillborn offspring were removed immediately. During the lactation period, the bedding was replaced as gently as possible to avoid excessive stress on the dams. After parturition, litter size, numbers of male and female mice, and the survival rates of the offspring were recorded for each cage.

Considering that the effects of menstrual periods of females may interfere with the effects of cage size in our later behavioral tests, only 160 male KM mice were randomly divided into four groups and raised in cages with two different sizes (see below).

To examine the effects of cage size upon mouse behavior, two groups of mice (standard cage vs. those from restrict cage) with two different age ranges (7 vs. 9 weeks old) were used in the OFT and the *T*-maze test, yielding four groups of mice (*n* = 40 for each group).

The OFT was utilized to examine exploration as well as anxious behavior. The test apparatus (XRXZ301, Shanghai Xinruan Information Technology Company Limited, China) consisted of a partially uncovered cube of 24 × 24 × 30 cm^3^ with commercial software for recording and analysis. The bottom floor was divided into a central area (12 × 12 cm^2^ at the very center) and a margin area (four squares of 6 × 6 cm^2^ in the corner). The OFT was performed to measure the spontaneous activities of mice as described by Sherif et al. (1994). The test room was dimly illuminated and quiet. A single mouse was placed in the same corner of the floor at each test session. Once tracking was acquired, which required approximately 30 s, each test lasted for 20 min. The time each mouse spent in the central or a margin part was recorded. A lower ratio of central/total residence time and decrease of the distance traveled within the central part are indications of increased anxiety-like behavior. The uncovered cube was cleaned with a 75% ethanol solution after each testing session.

A spontaneous alternation T-maze test (XRXT111, Xinruan Information Technology Company Limited, China) was used to measure the working memory of mice as previously described (Masuda et al., 1992; Mira et al., 2014). A T-maze was made of gray plastic consisting of three arms (one start arm and two open arms). Then the T-maze test was performed with 14 trials per day. In each trial, the test mouse always started from the start arm and was given a free choice to enter either of the open arms. Once the mouse entered one open arm, the other open arm was closed. If the open arm that the test mouse entered was different from its previous choice, for example, if the mouse chose to enter right arm on the first try and chose to enter left arm on the second try, this was considered an effective alternation. When a mouse had visited an open arm for 14 times, the test was terminated for that day. To better measure working memory, the same test was repeated for four consecutive days. The T-maze was cleaned after each testing session with a 75% ethanol solution.

### Statistical Analysis

To examine the effect of cage size on reproductive performance, delivery rate (i.e., the number of parturition dams divided by the number of total pregnant mice), litter number, survival rate, and male-to-female proportions were compared between the standard cage size group and restricted cage size group using an independent two-sample *t*-test.

To examine the effect of cage size on anxiety, a Cage (standard vs. restricted) × Age (7 vs. 9 weeks) two-way ANOVA on the anxiety level (i.e., the ratio of time in the margin areas to time in the center areas) was conducted. The Cage-related effects were of interest, and the *post hoc* comparisons were mainly focused on the differences between the standard cage size group and restricted cage size group for 7- and 9-week-old mice, using independent two-sample *t*-tests. Spearman tests were used to assess the correlation between residence time of a mouse in central area and distance traveled in the central part in each group.

To fully examine the effect of cage size on working memory, a Cage (standard vs. restricted) × Age (7 vs. 9 weeks) × Time (1st, 2nd, 3rd, and 4th test days) three-way repeated-measures ANOVA on working memory (i.e., the number of alteration visits) was conducted. Again, the Cage-related effects were of interest. When a significant effect was detected, a Tukey *post hoc* test was applied to identify the differences.

To examine whether anxiety and working memory were related, Spearman tests were used to assess the correlation between them at the 4th test day in each group.

The software *SPSS* 26.0 was used for statistical analysis. In addition, Origin 19.0 was also used for the graphics design. The descriptive statistical results were expressed as means ± standard deviation (SD). An alpha level of *P* ≤ 0.05 was considered significant for statistical inference.

## Results

### Restricted Cage Size Was Detrimental to Reproductive Performance

**Figure 1:**
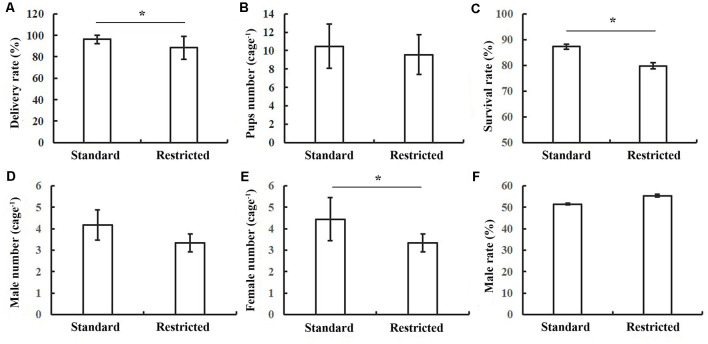
Statistical results of reproductive performance of mice under two conditions. **(A)** The delivery rate of mice was decreased in the restricted group. **(B)** No significant difference in pups’ number between two groups. **(C)** The survival rate of pups was decreased in the restricted group. **(D)** No significant difference in the survival male number of pups between two groups. **(E)** The survival female pups in restricted group were decreased. **(F)** No significant difference in survival male rate between standard and restricted groups. The error bars mean standard deviations. *P < 0.05, *n* = 26 per group.

The delivery rate was 96.15% and 88.64% for pregnant mice in standard cages and restricted cages, respectively; and this difference between the two groups was significant (*t*_(41)_ = 1.314, *P* = 0.049; Figure 1A). While the litter number was only marginally different between the two groups (*t*_(46)_ = 2.09, *P* = 0.07; Figure 1B), the survival rate of the offspring was significantly lower in the restricted cage group than in the standard cage group (*t*_(43)_ = 2.476, *P* = 0.023; Figure 1C). Particularly, the survival rate of female mice in the restricted cages showed a significant reduction when compared with that in the standard cages (*t*_(45)_ = 1.926, *P* = 0.014; Figure 1E), while the survival rate of the male did not differ between the two cage types (*t*_(43)_ = −1.703, *P* = 0.071; Figures 1D–F). The results were also summarized in Table 1.

**Table 1:** Reproductive capacity of mice in both cages.

	Standard	Restricted	*df*	*t*-stat	*P*-value
Delivery rate (%)	96.154 ± 13.856	88.462 ± 10.621	41	1.314	0.049
Litter number	10.48 ± 2.525	9.642 ± 2.147	46	2.09	0.09
Survival rate (%)	0.873 ± 0.096	0.798 ± 0.112	43	2.476	0.023
Female proportion (%)	0.486 ± 0.004	0.451 ± 0.071	45	1.926	0.014
Male proportion (%)	0.514 ± 0.043	0.553 ± 0.061	43	−1.703	0.071

*The data are mean ± standard deviation. *n* = 26 per group*.

### Restricted Cage Size Increased Anxiety and Decreased Exploratory Behavior

**Figure 2:**
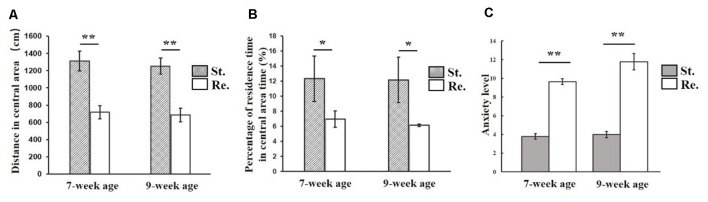
Cage effects on exploration and anxiety level of mice. **(A)** The distance traveling in central area decreased in restricted cage group; **(B)** the residence time in central area was decreased in restricted cage group; **(C)** the anxiety level of restricted cage group was increased significantly. The error bars mean standard deviations. Statistically significant differences compared with standard cage group. *P < 0.05, **P < 0.01, *n* = 40 per group. St., standard cage groups; Re., restricted cage groups.

The two-way ANOVA on the anxiety level showed a significant main effect of Cage (*F*_(1,155)_ = 1,285.52, *P* < 0.001, $\eta_{\text{p}}^{2}$ = 0.95). *Post hoc* comparisons revealed a significant increase in the anxiety level in the restricted cage groups for both 7-week-old (*F*_(1,78)_ = 8.653, *P* = 0.004, $\eta_{\text{p}}^{2}$ = 0.957) and 9-week-old (*F*_(1,78)_ = 366.975, *P* < 0.001, $\eta_{\text{p}}^{2}$ = 0.96) mice. Follow-up analyses showed significant Cage effects on time spent in the center and margin areas (*F*_(1,155)_ = 956.943, *P* < 0.001, $\eta_{\text{p}}^{2}$ = 0.796 and *F*_(1,155)_ = 1,470.262, *P* < 0.001, $\eta_{\text{p}}^{2}$ = 0.954, respectively). As shown in Table 2, mice from the restricted cage stayed longer in the margin areas than the mice from standard cage in both the 7-week (*t*_(78)_ = 16.67, *P* < 0.001) and 9-week cohorts (*t*_(78)_ = 18.357, *P* = 0.003). In contrast, the residence time in the central area was reduced in mice from the restricted cage compared with mice from the standard cage (*t*_(78)_ = −12.66, *P* = 0.023 and *t*_(78)_ = −8.344, *P* = 0.029 for the 7- and 9-week-old cohorts, respectively; Figure 2B). In addition, the traveling distance in the central part of mice from restricted cage was significantly shorter than that of standard-cage mice (*t*_(78)_ = −16.35, *P* < 0.001 and *t*_(78)_ = −13.736, *P* < 0.001 for the 7- and 9-week-old cohorts, respectively; Figure 2A). In each group, there was a positive correlation between distance traveled and residence time in the central area (*r* = 0.932, *P* < 0.001; *r* = 0.947, *P* < 0.001; *r* = 0.968, *P* < 0.001; and *r* = 0.956, *P* < 0.001 for 7-week standard, 7-week restricted, 9-week standard, and 9-week restricted, respectively), indicating a normal exercise ability of mice in each group. Together, these results suggested that the restricted cage size led to a decrease in exploratory behavior and to an increase in anxiety in KM mice. The results of the ANOVA and *t*-test are listed in Tables s 3, 4 respectively.

**Table 2:** The results of OFT and *T*-maze tests.

		Standard cage	Restricted cage	Standard cage	Restricted cage	
		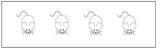	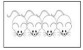	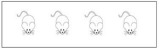	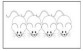
	Behavioral test	7 weeks old		9 weeks old	
OFT	Distance in central area (cm)	1,312.67 ± 114.22	732.54 ± 80.31	1,279.85 ± 96.27	699.28 ± 81.84
	Time in central area (%)	12.33 ± 3.02	6.96 ± 0.10*	12.12 ± 3.01	6.13 ± 0.11*
	Time in margin area (%)	46.10 ± 4.37	67.51 ± 9.78**	47.92 ± 4.46	72.68 ± 10.83**
*T*-maze	1st day	6.21 ± 0.96	7.31 \pm 1.22	5.71 ± 0.94	7.03 ± 1.82
	2nd day	7.14 ± 1.05	8.38 ± 1.78*	6.14 ± 1.55	7.78 ± 2.02
	3rd day	7.76 ± 0.98	8.52 ± 1.19	8.26 ± 1.32	9.32 ± 1.87
	4th day	8.38 ± 0.89	9.45 ± 1.26	8.88 ± 1.29	10.62 ± 1.52*

The data are mean ± standard deviation. Statistically significant differences compared with standard cage group, *P < 0.05, **P < 0.01 (*n* = 40 per group). OFT, open-field test.

**Table 3:** Results of ANOVA for OFT and *T*-maze test.

	Anxiety level				Working memory			
	Two-way ANOVA				Three-way repeated-measures ANOVA			
	*df*	*F*	*P*-value	$\eta_{\text{p}}^{2}$	*df*	*F*	*P*-value	$\eta_{\text{p}}^{2}$
Cage	1,156	1,285.52	<0.001	0.95	1,154	12.676	<0.001	0.075
Age	1,156	94.644	<0.001	0.376	1,154	2.115	0.148	0.013
Cage × Age	1,156	98.177	<0.001	0.39	1,154	0.721	0.168	0.007
Time					3,154	288.632	<0.001	0.648
Cage × Time					3,154	0.436	0.67	0.003
Age × Time					3,154	4.581	0.008	0.028
Cage × Age × Time					3,154	0.247	0.21	0.01

*n = 40 per group. OFT, open-field test*.

**Table 4:** The *post hoc* *t*-tests for OFT and *T*-maze ANOVAs.

Test	Groups	*df*	*t*-stat	*P*-value
OFT	7-week central	78	−12.66	0.023
	7-week margin	78	16.67	<0.001
	9-week central	78	−8.344	0.029
	9-week margin	78	18.357	0.003
*T*-Maze	7-week 1st	78	−1.034	0.061
	7-week 2nd	70	−2.341	0.021
	7-week 3rd	78	−1.066	0.059
	7-week 4th	77	−1.093	0.06
	9-week 1st	78	−1.102	0.078
	9-week 2nd	77	−1.105	0.052
	9-week 3rd	59	−1.017	0.07
	9-week 4th	72	−2.759	0.03

*n = 40 per sample. OFT, open-field test*.

### Restricted Cage Size Increases Working Memory

**Figure 3:**
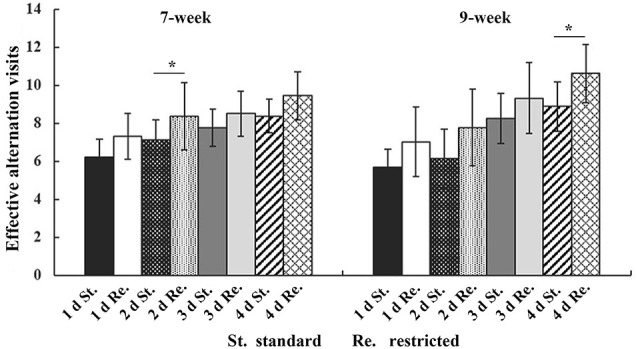
The effective alternation visits of mice in *T*-maze test. The error bars mean standard deviations. Statistically significant differences compared with standard group. *P < 0.05, *n* = 40 per group. St., standard cage groups; Re., restricted cage groups.

In three-way repeated-measures ANOVA on working memory, significant main effects were found for Cage (*F*_(1,154)_ = 12.676, *P* < 0.001, $\eta_{\text{p}}^{2}$ = 0.075) and Time (*F*_(3,154)_ = 288.632, *P* < 0.001, $\eta_{\text{p}}^{2}$ = 0.648), but not for Age (*F*_(1,154)_ = 2.115, *P* = 0.148, $\eta_{\text{p}}^{2}$ = 0.013). A significant interaction between Time and Age was also detected (*F*_(3,154)_ = 4.581, *P* = 0.008, $\eta_{\text{p}}^{2}$ = 0.028). In *post hoc* analyses, mice from the restricted cages showed higher working memory scores on each test day, but it was only at the second test day for the 7-week-old groups (*t*_(70)_ = −2.341, *P* = 0.021) and at the 4th test day for the 9-week groups (*t*_(72)_ = −2.759, *P* = 0.03) that the cage effects on working memory were statistically significant (Figure 3, Table 2). Furthermore, an obvious increase of working memory according to test days could be seen in each group (repeated-measures ANOVA showed the smallest *F* = 16.443, *P* < 0.001, $\eta_{\text{p}}^{2}$ = 0.936). The results of ANOVA and *t*-test are shown in 3, 4, respectively.

### Cage Size May Impact Anxiety and Working Memory Independently

**Figure 4:**
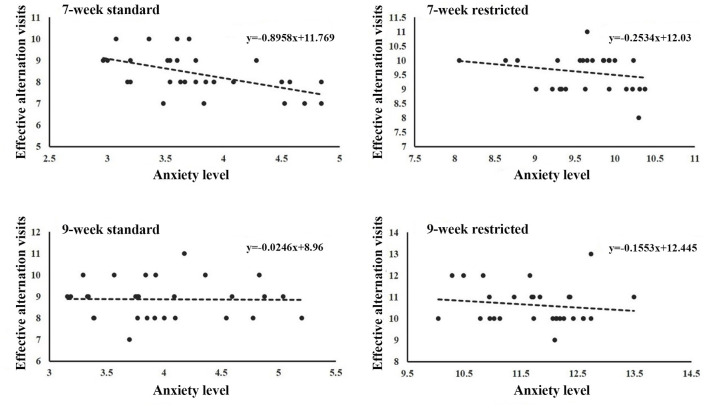
The relationship between anxiety and working memory. A significant correlation of anxiety level with working memory was only found in the 7-week-old standard-cage mice (*P* < 0.01), but not other groups.

Since mice from the restricted cages showed higher working memory scores and higher anxiety measures than mice from the standard cages, one may conjuncture that cage size could have an impact on working memory and anxiety in a dependent way. We therefore explored the correlation between the anxiety level and working memory in each group. As is shown in Table 4, Figure 4, the anxiety level had a negative correlation to working memory at the fourth test day only in the 7-week-old mice from the standard cages (*r* = −0.526, *P* = 0.003) but not in mice from the restricted cages (Figure 4, Table 3), suggesting that the effects of cage size on working memory are independent from its effect on anxiety.

## Discussion

We found that the reproductive performance, exploration activity, anxiety level, and working memory of KM mice are affected by cage size. Restricted cages can increase miscarriage rate of dams and decrease offspring survival rate, especially of female pups. Further, restricted cage size causes an increase in anxiety levels and an improvement in working memory. As discussed below, these results provide empirical evidence for improving the welfare of KM mice, with some cautions about the effects of cage size on behavioral tests.

The size of living space and group size are important parameters for the physical and mental health of animals. A crowded living space could cause unexpected effects on animal behavior and cognitive function (Kuipers et al., 2013). However, physiological and psychological problems caused by space environment can be easily ignored. Our data suggest that the pregnant mice are more likely to have a miscarriage in the restricted cages and that the survival rate of their offspring is significantly lower than that of standard cages. In contrast to our study, Whitaker et al. (2009) assessed reproductive performance by comparing the number of pups born and the survival rate between standard cages and larger cages. They found that exceptionally large cages may be detrimental to breeding performance. Taken together, the effects of cage size on reproductive performance are complex and may have an inverted U-shape. More studies are warranted to determine the optimal cage and group size, which might differ for each strain of mice. Regarding the decrease of reproductive performance in restricted cages, we found that more female pups were hurt by their mother mouse in the restricted cages. We speculate that this is probably a regulation strategy controlling group size when the living space is restricted. Considering the higher anxiety levels observed in mice from the restricted cage (Figure 2C), it is also possible that poor reproductive performance is, at least partially, caused by unwell emotional states such as anxiety. Although breeding density varies proportionally with cage size for a certain group size, previous reports (Loo et al., 2001; Bellei et al., 2011; Buijs et al., 2011) did not find significant differences in reproductive performance between two groups of mice with different breeding densities (10 and 20 mice per cage, respectively) in the same cage with the size of (34 × 49 × 16 cm^3^). The anxiety levels also showed no significant difference between the two groups. However, there appeared to be an interactive effect between cage size and animal raising density for the effects on both reproduction and behavior.

We found higher levels of anxiety in mice raised in the restricted cages. This is consistent with findings from a previous physiology study (Barnett et al., 1992; Gupta et al., 2007; Villagrá et al., 2009) showing that, compared with animals raised in regular cages, animals raised in small cages have higher levels of corticosterone, a biological indicator for stress levels. We believe that decreased exploration behavior and increased anxiety levels of the mice in this study are the manifestations of a stress response. This result indirectly proved that being confined to a small space is likely to stimulate the stress response of the animals and trigger anxiety, which could be a key factor leading to increased abortion rate of pregnant mice in restricted cages.

Here, we found that mice from the restricted cages showed better working memory as assessed with the T-maze test. This result seems to conflict with findings from a previous study (Klein, 2002) that showed that working memory capacity is diminished in people undergoing stressful life events. In contrast, a study in humans reported that participants were in significantly better moods after viewing a comedy routine, while the mood of a control group had not changed. When both groups were then given a memory test, the result showed that people with a better emotional mood have lower working memory. These studies indicate that both positive and negative mood states may adversely affect working memory. Indeed, research results have shown that the influence of stress levels on cognitive ability has an inverted U shape—therefore, individuals perform better under a moderate stress level than they do without stress or with too much stress (Koob, 1991; Luethi et al., 2008; Martin and Kerns, 2011; Steenbergen et al., 2015). This may explain why we observed better working memory in the restricted cage groups despite their higher anxiety level. In addition, a previous study found a negative correlation between the anxiety level and working memory (Klein, 2002). In our study, such a negative association was only observed in 7- but not 9-week-old mice from the standard cages, which suggests that the influence of anxiety on working memory is age dependent. Furthermore, the lack of association between anxiety and working memory in the mice from the restricted cages might indicate that cage size can impact anxiety and working memory independently.

Although it is widely known that a restricted space is more likely to cause physical and mental problems, this is often overlooked due to insufficient understanding of the correlation between environmental stress and these problems. The present study suggests that restricted space is detrimental to the physical and mental health of adolescent and early adult mice. We have not yet explored if there is a similar effect on middle-aged and old mice. As mental abilities vary with age in humans (Deary et al., 2000), it is reasonable to expect that the stress endurance for mice at different ages might be different as well. Taking into account the influences of the menstrual period of female mice, we only evaluated the effects of cage size on the behaviors of male mice in the OFT and T-maze tests. In addition, as mice have very high drives to build nests (Van de Weerd et al., 1998; Deacon, 2006), they may still experience nesting-related stress because the tissue articles we provided may not satisfy their needs to build nests in a limited space in both types of cages.

In conclusion, in the present study, we found that size-restricted cages have a detrimental effect on the reproductive performance, exploratory behavior, and anxiety of KM mice but increase their working memory performance in a T-maze test. Future studies using KM mice should be mindful of the effect of the housing environment, particularly the size of the cage, on reproduction and behavioral tests.

## Data Availability Statement

The original contributions presented in the study are included in the article, further inquiries can be directed to the corresponding author/s.

## Ethics Statement

The animal study was reviewed and approved by Animal Care and Use Committee of the animal facility at Northwest Normal University.

## Author Contributions

All authors contributed to the article and approved the submitted version.

## Conflict of Interest

The authors declare that the research was conducted in the absence of any commercial or financial relationships that could be construed as a potential conflict of interest.
